# Correction: Exogenous application of moringa leaf extract improves growth, biochemical attributes, and productivity of late-sown quinoa

**DOI:** 10.1371/journal.pone.0262980

**Published:** 2022-01-19

**Authors:** The PLOS ONE Staff

In the Funding section, the grant number from the funder King Saud University is listed incorrectly. The correct grant number is: RSP-2021/193. The publisher apologizes for the error.
